# From positive projection to delirium. About a case

**DOI:** 10.1192/j.eurpsy.2024.1562

**Published:** 2024-08-27

**Authors:** M. V. Barea, L. S. Rodriguez, S. C. Bailen

**Affiliations:** SAS, Jaen, Spain

## Abstract

**Introduction:**

Erotomania, was described in 1942, is more common in women than in men, although the incidence is unknown. This syndrome is usually characterized by a young woman with the illusion that a man whom she considers to be of a higher social or professional position is in love with her. Developing an elaborate delusional process about this man, his love, his pursuit and total commitment to the idea. Two forms, pure or secondary, are described. As well as fixed or recurring

52-year-old female patient in outpatient follow-up with a diagnosis of schizophrenia with long-term follow-up, start of follow-up by a new therapist, in this context intensive follow-up is carried out in the event of the appearance of pharmacological secondary effects, pharmacological readjustment is carried out with good results.

During the joint follow-up with nursing, the cessation of secondaryisms is confirmed and we are informed of the gradual appearance of overvalued ideas in relation to the new therapist, which are gradually structured in the form of erotomanic delirium that coincides with the cessation of follow-up by said therapist. Consultations in the emergency room occur on a couple of occasions due to mild behavioral alterations secondary to messages and communications that he reports receiving where said love is confirmed. Despite readjustments, there continues to be an increase in clinical symptoms due to abandonment of medication, finally producing serious alterations aimed at the search for said therapist, finally culminating in admission to the acute care unit for containment of said condition.

**Objectives:**

The objetives is the diferencial diagnosis, in this case symptoms could be classified as positive symptoms of schizophrenia, although it is its own nosological entity.

**Methods:**

.

**Results:**

.

**Conclusions:**

This patient represents a classic example of De Clerambault syndrome and is a faithful expression of the recurrent syndrome associated with delusions of grandeur, eroticism and jealousy. There have also been ideas of reference and agitated behavior associated with his delusional process.

**Disclosure of Interest:**

None Declared

